# Media studies to enhance the production of verticillins facilitated by in situ chemical analysis

**DOI:** 10.1007/s10295-018-2083-8

**Published:** 2018-09-27

**Authors:** Chiraz Soumia M. Amrine, Huzefa A. Raja, Blaise A. Darveaux, Cedric J. Pearce, Nicholas H. Oberlies

**Affiliations:** 1 Department of Chemistry and Biochemistry, University of North Carolina at Greensboro, P.O. Box 26170, Greensboro, NC 27402 USA; 2 Mycosynthetix, Inc., 505 Meadowlands Drive, Suite 103, Hillsborough, NC 27278 USA

**Keywords:** Verticillin, Epipolythiodioxopiperazine alkaloids, In situ extraction, Filamentous fungi, Fermentation

## Abstract

**Abstract:**

Verticillins are a group of epipolythiodioxopiperazine alkaloids that have displayed potent cytotoxicity. To evaluate their potential further, a larger supply of these compounds was needed for both in vivo studies and analogue development via semisynthesis. To optimize the biosynthesis of these secondary metabolites, their production was analyzed in two different fungal strains (MSX59553 and MSX79542) under a suite of fermentation conditions. These studies were facilitated by the use of the droplet-liquid microjunction-surface sampling probe (droplet probe), which enables chemical analysis in situ directly from the surface of the cultures. These experiments showed that the production of verticillins was greatly affected by growth conditions; a significantly higher quantity of these alkaloids was noted when the fungal strains were grown on an oatmeal-based medium. Using these technologies to select the best among the tested growth conditions, the production of the verticillin analogues was increased while concomitantly decreasing the time required for fermentations from 5 weeks to about 11 days. Importantly, where we could previously supply 5–10 mg every 6 weeks, we are now able to supply 50–150 mg quantities of key analogues per month via laboratory scale fermentation.

**Graphical abstract:**

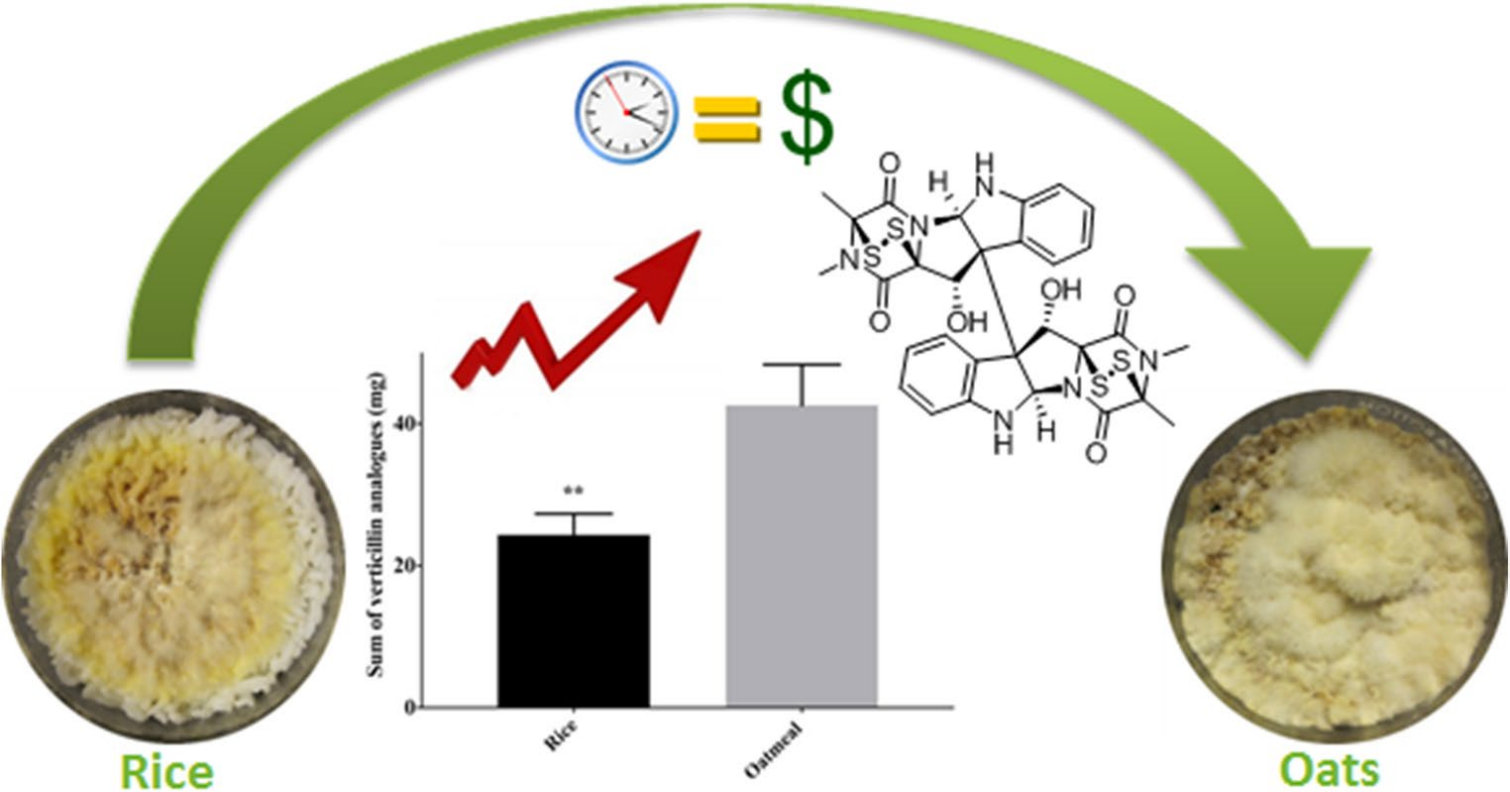

**Electronic supplementary material:**

The online version of this article (10.1007/s10295-018-2083-8) contains supplementary material, which is available to authorized users.

## Introduction

Fungi are a rich resource for biologically active and structurally diverse secondary metabolites [16, 26, 27]. As such, a number of authors have noted the value of investing in fungi as a source of new drug leads, especially to treat cancer and overcome its resistance [6, 44]. Cancer is a worldwide problem, and in the USA alone, over one million new cases were estimated for 2016, resulting in more than half a million cancer-related deaths [70].

As part of ongoing studies to discover new anticancer leads from filamentous fungi [20, 44, 55], our team has isolated hundreds of fungal metabolites over the last decade [16]. Of these, verticillins are one of the most interesting for a variety of reasons [20, 48, 49, 58, 79]. This class of bioactive fungal secondary metabolites (as exemplified by compounds **1–7**) is characterized by a dimeric structure [24] and is a key member of the epipolythiodioxopiperazine (ETP) alkaloids. Biologically, these compounds display potent antitumor activity [10, 11, 35, 72, 80], along with antibacterial [36, 53, 81, 82], nematicidal [14] and immune induction properties [18].

The lead compound of this class, verticillin A (**1**), was first discovered in 1970 [37], and since then, 27 verticillin analogues have been characterized [20]; even 30 years ago, their cytotoxic potential was noted [35]. At the time of discovery, verticillin A (**1**) showed an ED_50_ of 0.2 µg/mL (0.27 µM) against HeLa cells [37]. In another study, verticillin A (**1**), along with the closely related analogues, Sch 52900 (**5**) and Sch 52901 (**2**), showed potential in preventing the transcription of *c*-*fos* promoter gene, which is a proto-oncogene found overexpressed in a variety of cancers [12]. Two analogues, 11, 11**ʹ**-dideoxyverticillin A (**6**) and 11**ʹ**-deoxyverticillin A (**7**), demonstrated in vitro cytotoxicity against HCT-116 (human colon carcinoma) cells in the nanomolar range [72]. These later compounds also demonstrated an inhibitory effect of tyrosine kinase on the growth factor receptor, thereby leading to anti-tumor activity [80]. Verticillin A showed potent activity in sensitizing human colorectal cancer cells to apoptosis both in vitro and in vivo, as well as, the ability to induce cell-cycle arrest at the G_2_ phase [48].

Biologically, interest has been growing in the verticillins, particularly after verticillin A (**1**) was shown to be a selective histone methyl transferases (HMT) inhibitor [58]. The HMT inhibitory activity resulted in the demethylation of H3K9me3 of the silenced FAS gene [58], thereby allowing its transcription, such that FAS was re-expressed, triggering apoptosis mediated cell death [45]. Moreover, verticillin A (**1**) induced apoptosis selectively against malignant tumor cells, while minimal effect was exhibited against healthy cells [79]. Verticillin A (**1**) showed potential to overcome metastatic colon carcinoma resistance to 5-fluorouracil. Mice transplanted with SW620-5FU-R cells were treated with a combination of verticillin A (**1**) and 5-fluorouracil and displayed significantly smaller tumors compared to the groups receiving only one of these two treatments [58]. In a recent study conducted on pancreatic cancer by Lu et al. [49], verticillin A was given to two groups of mice transplanted with either PANC02-H7 or UN-KC-6141 cells. Verticillin A was administrated alone or in combination with anti PD-L1 (programmed death ligand) treatment to provide immunotherapy. The results demonstrated that verticillin A (**1**), in conjugation with anti PD-L1 ligand, markedly reduced tumor size and viability. In summary, a suite of pharmacological studies over the last 5 years has increased the interest surrounding this class of compounds.

Despite the promising activities that verticillins have shown in vitro and in vivo, research has been hindered by the lack of material. In previous work, 0.66–1.72 mg of verticillin analogues (**1–3**) per g of extract could be isolated from large scale fermentations of fungal cultures containing 150 g of rice (with a total isolated of 1.6–5.5 mg) [20]. Similarly, 0.37–0.85 mg of verticillin analogues were isolated per g of extract (with a total isolated of 3.5–8.0 mg) [36]. Based on the literature, the yields of these compounds were not improved greatly when grown in liquid cultures. For example, from a 73 L fermentation, the yield of verticillin G was 0.58 mg per g of extract (with a total isolated of 2.9 mg) [82]. In another study that used 20 L fermentations, compounds **1**, **6** and **7** were isolated with a yield of 0.25 mg, 0.61 mg and 1.14 mg per g of extract, respectively (with a total yield of 3.5 mg, 8.5 mg and 16 mg, respectively) [72]. Furthermore, although elegant synthetic approaches have been reported for the preparation of various epipolythiodioxopiperazine alkaloids [8, 42, 43], these processes require numerous reactions steps, making the large-scale production of these compounds challenging. About 0.2 g of (+)-11,11**ʹ**-dideoxyverticillin A was prepared in 14 steps from ˃ 10 g scale of commercially available amino acids derivatives [42]. In summary, the production of the verticillins in the literature, whether using liquid-based or solid-based fermentations, has been largely on the scale of only a couple mg, and most of the verticillins have never been synthesized. For these reasons, the adequate supply of key analogues must be addressed before the full pharmacological potential of these compounds can be realized.

Many studies have shown the influence of media and environmental factors on fungal biosynthesis [4, 9], where optimal nutrition is required for ideal fungal growth [46, 51]. The OSMAC approach is a prominent strategy to probe the importance of fungal fermentation conditions, potentially generating broader chemical diversity [29, 30] and/or targeting the enhanced production of a distinct class of secondary metabolites. In this study, several fungal strains were selected from the Mycosynthetix library. These cultures were grown on a suite of different solid media, and productivity was assessed by taking advantage of new instrumentation that facilitates the screening of the chemistry of the cultures. The droplet-liquid microjunction-surface sampling probe (droplet probe) permits the in situ analysis of targeted secondary metabolites directly from the surface of cultures [40, 67]. Our goal was to enhance the biosynthesis of verticillins by identifying a strain that produces the highest quantity of the analogues under appropriate fermentation conditions in the shortest period of time.

## Materials and methods

### Mycology

Fungal cultures were provided by Mycosynthetix, Inc., via an ongoing project to identify diverse natural product study material as a source of anticancer drug leads [44]. Over the last decade, we have analyzed the chemistry and biological activity of thousands of cultures from the Mycosynthetix library, and among these, ten cultures were identified as being able to biosynthesize verticillin A and its analogues (compounds **1–5**) based on published dereplication protocols [15, 56]. Accordingly, the following strains were chosen for this study: MSX74391, MSX71844, MSX58124, MSX75296, MSX75281, MSX45374, MSX70777, MSX59553 and MSX79542 (Table 2S). In a previous study, fungal strains MSX59553 and MSX79542 were both identified as *Clonostachys rogersoniana* [56].

### Identification of fungal strains

The different MSX strains utilized in the present study were identified to the genus and/or species level using the fungal ITS region (ITS1–5.8S-ITS2) of the nuclear ribosomal operon, which was amplified with primer combination ITS5 and ITS4 with primers ITS5/ITS1F and ITS4 [23, 77]. Methods for BLAST search in NCBI GenBank and Maximum Likelihood phylogenetic analysis, as utilized to identify fungal strains, have been detailed recently [61]. The BLAST search and phylogenetic analysis using taxon sampling [1] revealed that MSX59553, MSX79542, MSX74391, MSX58124, MSX75296, MSX75281, MSX45374, and MSX70777 belong to the genus *Clonostachys* (Ascomycota, Hypocreales, Bionectriaceae), while MSX71844 was identified as *Purpureocillium lavendulum* (Ascomycota, Hypocreales, Ophiocordycipitaceae) (Fig. S1). Phylogenetic analysis using the ITS region also showed that strains MSX59553, MSX45374, MSX74391, MSX79542, MSX70777, and MSX58124 had phylogenetic affinities with *C. rogersoniana* [64]. Our results were in agreement with a recent genomic study identifying the biosynthetic gene clusters for verticillins in *C. rogersoniana* [76]. As the ITS region alone is not informative to identify species in certain orders of the Ascomycota, including Hypocreales [33], future taxonomic and molecular phylogenetic studies will incorporate sequence data from protein-coding regions to more precisely identify species names for verticillin-producing strains [54]. The sequence data were deposited in GenBank (accession numbers: KX845687, KX845688, MH421853, MH421854, MH421855, MH421856, MH421857, MH421858, MH421859, and MH421860).

For verticillin producing strains, there has been some confusion on the fungal taxonomy [13]. Earlier studies on the chemistry of verticillin analogues have identified the verticillin-producing fungi as belonging to the genera *Gliocladium, Penicillium,* and *Verticillium* [36, 37, 72]. The morphology of the flask shaped phialide in all three genera can be a cause of confusion for non-mycologists. Moreover, fungal taxonomic names have been rapidly changing due to the use of molecular sequence data in the past 20 years [61]. In addition, the name *Bionectria* (sexual state) has been used previously in the literature for a fungal strain producing verticillin G [82]; while this was accurate, the use of name *Bionectria* is now phased out due to the adoption of One Fungus = One Name [28, 73], in accordance with the recent changes concerning pleomorphic fungi in the *International Code of Nomenclature for algae, fungi, and plants*. For pleomorphic names (sexual and asexual) in the family Bionectriaceae, Rossman et al. [63] have proposed the use and protection of the asexual morph (*Clonostachys*) rather than the sexual morph (*Bionectria*). Despite the adoption of the name *Clonostachys*, the subgenus *Bionectria* is still being used in the literature. Based on our recent identification of fungal strains that make verticillin and its analogues, we have identified two fungal genera, *Clonostachys* spp. and *Purpureocillium lavendulum* [59], that biosynthesize the epipolythiodioxopiperazine-type alkaloids.

### Media and fermentations

For the analysis of fungal cultures in Petri dishes, seven types of agar media were used, and ingredients to make these were purchased from Difco, ACROS, and Research Products International (RPI): malt extract agar, potato dextrose agar, yeast extract soy peptone dextrose agar, Spezieller Nährstoffarmer agar, Sabouraud dextrose agar, potato dextrose mushroom and oatmeal agar (See Table 1S for compositions). A small piece (~ 0.5 cm^2^) of fresh culture grown on potato dextrose agar was cut from the leading edge of 2-week-old colony from strains MSX59553 and MSX79542 and was used to inoculate various types of agar media in duplicate, respectively. The plates were then incubated at room temperature until the cultures showed good growth as noted by the expansion of the colony over the surface of the medium (~ 3 to 4 weeks).

Rice fermentations [(1:1 commercial white rice (variety: Calrose Botan; 5 g) and (variety: Sona Masoori; 5 g)] were prepared by adding 10 g of rice to a Petri dish or a 250 mL flask with 25 mL of DI-H_2_O, followed by autoclaving at 221 °C for 30 min. Oatmeal media (Old fashioned Quaker oats) was prepared using 10 g of rolled oats and ~ 17 mL of DI-H_2_O, followed by autoclaving at 221 °C for 30 min. The flasks containing the rice or oatmeal media were inoculated with a seed culture grown in 10 mL of YESD broth media (2% soy peptone, 2% dextrose and 1% yeast extract) for about 5 days with agitation (100 rpm) at room temperature. The inoculated cultures were grown at room temperature for ~ 4–5 weeks, except for the time progression studies, where each duplicate of the oatmeal culture was extracted every few days, from days 2 to 35. Strains were scaled-up in a similar way, using 2.8 L Fernbach flasks (Corning, Inc., Corning, NY, USA). The medium was prepared using 150 g of either rice or oatmeal, with 300 mL or 250 mL of DI-H_2_O added for rice medium and oatmeal medium, respectively, and both sterilized at 221 °C for 30 min. In addition, and as noted in more detail in the supplement (Fig. S7), augmenting the oatmeal media with amino acids was also examined. However, this did not result in significant differences in the biosynthesis of verticillins, and thus, this line of inquiry was not pursued further.

### Extraction of fungal cultures

Each culture was chopped with a spatula to ensure thorough extraction. For the cultures grown on Petri dishes, the chopped pieces were transferred into 150 mL flasks. Solutions of 60 mL of 1:1 chloroform:methanol (CHCl_3_:MeOH) were poured in each flask, followed by overnight shaking (100 rpm) at room temperature. The cultures were filtered by vacuum, and then the filtrates were mixed with 90 mL of CHCl_3_ and 150 mL of H_2_O and stirred for 30 min. The mixtures were transferred into a separatory funnel, where the bottom organic layer was evaporated in vacuo and reconstituted with 100 mL of 1:1 acetonitrile:methanol (CH_3_CN:MeOH) and 100 mL hexanes. Again, the defatted organic layers were collected and evaporated in vacuo.

### In situ analysis of fungal metabolite profiles via the droplet probe coupled with UPLC-PDA-HRMSMS/MS

The use of the droplet probe with fungal cultures has been detailed previously [67] and several examples have been published [57, 65, 66, 68, 69]. A CTC/LEAP HTC Pal auto-sampler was converted to a droplet probe with assistance from colleagues at Oak Ridge National Laboratories [38, 39]. Microextractions (3–5 µL) were performed on precise spots directly on fungal cultures using a 1:1 solution of MeOH:H_2_O. The extractions were injected onto a UPLC, and the chromatographic method followed previous protocols [15] used in the dereplication of fungal extracts. A Waters Acquity UPLC system was used with a BEH C_18_ column (Waters; 50 mm × 2.1 mm × 1.8 µm) heated to 40 °C and a mobile phase using a gradient from 15 to 100% CH_3_CN; the other solvent was 0.1% formic acid-H_2_O. The run utilized a flow rate of 0.3 mL/min for 10 min. UV data were collected from 190 to 500 nm and the eluent was split into a Thermo Fisher Scientific Q Exactive Plus mass spectrometer via electrospray ionization (ESI). MS data were collected from 150 to 2000 *m*/z at a resolution of 70,000 while alternating between positive and negative modes. MS/MS was performed with an HCD value of 35, and Xcalibur software was used for the primary analysis.

### Analysis of fungal metabolite profiles via UPLC-PDA-HRMSMS/MS

**Fig. 1:**
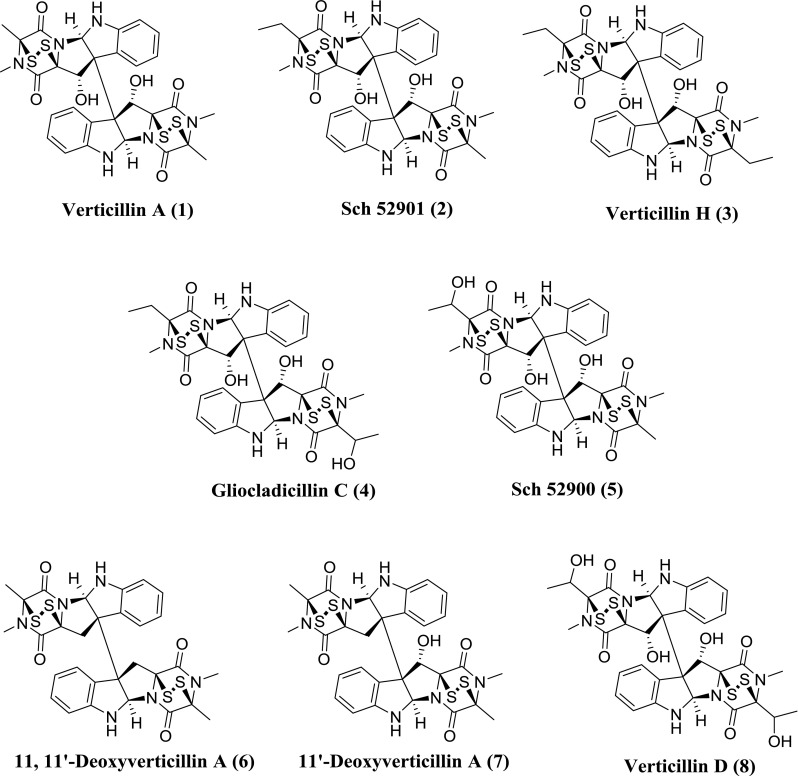
Structures of verticillin A and related analogues. Compounds **1–5** were the target for the biosynthetic optimization studies

Extracts of the plates and flasks were dissolved separately in 1:1 MeOH:dioxane to elaborate concentrations of 2.5 × 10^−2^, 0.5 or 2.0 mg/mL; 3 μL were injected directly onto the UPLC-PDA-HRMSMS/MS system. In addition to HRMS and MSMS data, retention times and UV data were used as mutually supportive data. The relative peak areas of each targeted secondary metabolite were measured (Fig. 1).

## Results and discussion

### Different fungal strains biosynthesize distinct patterns of verticillin analogues

**Fig. 2:**
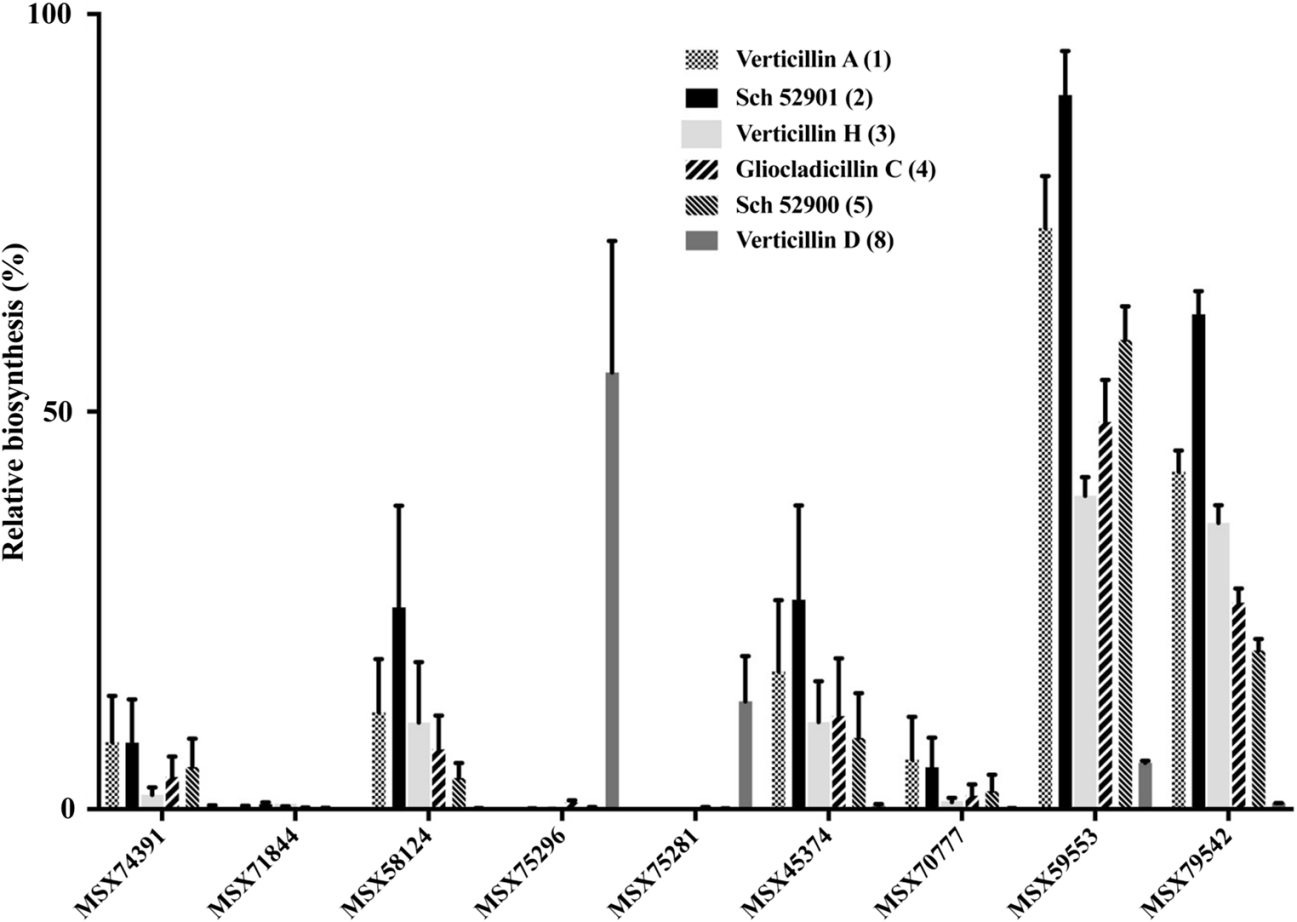
Relative production of various verticillin analogues grown on rice for 4 weeks across ten different fungal strains as measured by LC-HRMS in three replicates. Strains MSX59553 and MSX79542 demonstrated the highest relative biosynthesis of the targeted verticillin analogues (compounds **1–5**). Interestingly, specific strains (such as MSX75296 and MSX75281) showed the biosynthesis of higher amounts of verticillin D (**8**), corresponding to low to minimal production of the other analogues

The dereplication protocol was used to identify fungal strains that produced verticillins [15] (Table 2S Supporting Information). Extracts of suspected verticillin producing strains from our library were screened using UPLC-HRMS, revealing a higher production of verticillin analogues within the organic extracts of both strains MSX59553 and MSX79542 (Fig. 2). The graph shows the relative percentage of the targeted verticillin analogues as measured in triplicate. To more accurately compare the total production of the secondary metabolites, the calculations were normalized to the largest value and multiplied by the total mass of the defatted organic extract of each strain. Interestingly, some strains produced higher amounts of verticillin D (**8**) but only low levels of verticillin A (**1**) and related analogues (and vice versa), suggesting that the biosynthetic pathway for these analogues were somewhat different, even though the fungi were presumably all using non-ribosomal peptide synthesis to produce these metabolites [21].

### Use of the droplet probe for in situ chemical analysis to optimize the production of verticillins

Optimization of fermentation parameters is a well-established approach to enhance the yield of compounds biosynthesized by microorganisms [41, 78]. The alteration of media parameters had an important role in varying the biosynthesis of secondary metabolites in numerous fungal strains [7, 19, 22, 52]. In most cases, investigations of optimal fermentation conditions are targeted for specific active compounds, such as antibiotics [2, 60, 71].

**Fig. 3:**
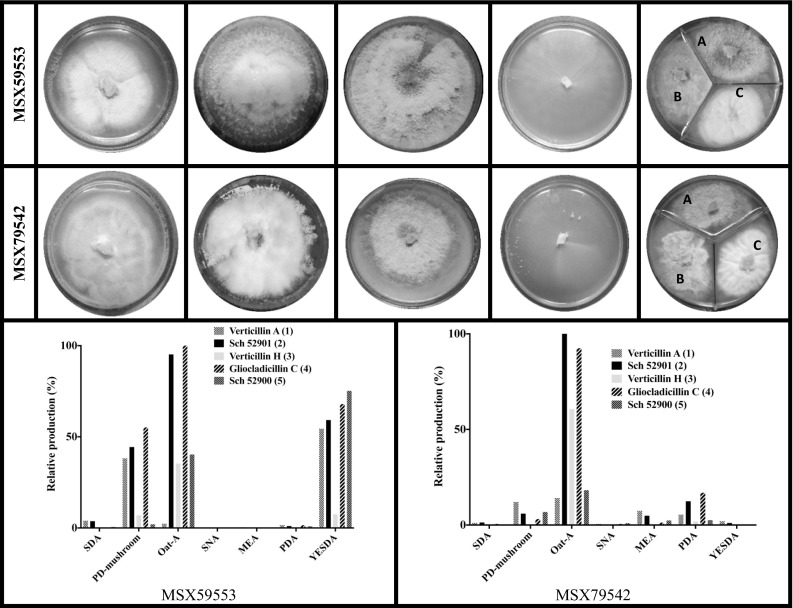
Top: Photos of strains MSX59553 (top) and MSX79542 (bottom). These fungal cultures were grown on SDA, PD-mushroom, Oat-A, SNA and PDA(**A**)/MEA(**B**)/YESDA(**C**) (from left to right, respectively) for 4 weeks. Fungal growths were morphologically distinct based on media, and guttates were observed only on cultures grown on PD-mushroom and Oat-A. Bottom: Relative biosynthesis of verticillin analogues via droplet probe directly from the surface of the strains MSX59553 (bottom left) and MSX79542 (bottom right)

Toward this end, the variation of culture media showed the most immediate results. A visual difference in the growth of the two fungal strains (MSX59553 and MSX79542) was noted (Fig. 3). Guttates, which we and others hypothesize concentrate secondary metabolites [25, 34, 66, 75], were noted when these were grown on Oat-A, YESDA, and PD-mushroom agar (Fig. S6), but not found on the fungi grown on the other nutrient media.

During this study, solid media were chosen over submerged liquid media, despite the advantages of the latter in offering control over the fermentation parameters and the simplification of scale-up for industrial purposes [50]. Pragmatically, we do not have the facilities for large-scale liquid fermentation, and moreover, liquid media for the production of verticillins did not show promise in the literature [12, 72, 82]. Furthermore, the chemistry of the cultures grown on solid media could be studied readily in situ via the droplet probe. In addition, solid fermentation is closer to the natural habitat of fungi, offering the mycelium a support for growth [62] and permitting better oxygen circulation than can be achieved by the high viscosity of liquid fermentation [17]. Previous comparative studies showed a notably higher productivity of pure compounds when microorganisms were grown on solid media, in comparison to liquid cultures [2, 3, 5, 32, 47, 74] with shorter fermentation time and less risk of enzymatic degradation [31].

The in situ microextractions by droplet probe were analyzed by UPLC-HRMS to identify the targeted verticillin analogues in each culture. Previously, a dereplication strategy [67] demonstrated the effectiveness of direct identification of secondary metabolites from cultures grown in Petri dishes. The peak areas of targeted compounds were averaged to build graphs (Fig. 3). For each culture condition, three sampling points were acquired on duplicate plates (*n* = 6). However, error bars were not calculated for two reasons. First, the three different spots represent different fungal growth stages, essentially oldest to youngest when sampling radially from the center. Also, the fungal growth morphology on each media had different traits, such as guttates (liquid droplets) or mycelium (filamentous hyphal growth). Due to this inherent biological variability, the recovery of the 5 µL droplet during microextraction was not always precisely the same (Fig. S2).

The targeted secondary metabolites (Fig. 1) were eluted within a retention time windows of 5.30–6.30 min on the 10 min gradient method. Parameters such as retention time, UV profile, mass range within 5 ppm, and fragmentation patterns were used to identify the structurally related analogues [56]. The production of these compounds was compared by measuring the percentage of the relative production of each compound normalized to the largest value. The biosynthetic profile varied according to the respective growth media. Both strains showed essentially zero to slight production of verticillin analogues in SDA, SNA, MEA and PDA media. Interestingly, a recent study on the gene clusters of verticillin biosynthesis noted PDA as a condition where the genes for biosynthesis were not expressed [76]; our phenotypic data (i.e., secondary metabolites) were supportive of their genotypic data. Of all the conditions examined, Oat-A was the most productive for verticillin biosynthesis in MSX79542 (Fig. 3). Similar results were observed with Oat-A in strain MSX59553, although two other media also showed promise (YESD and PD-mushroom).

### Production of the targeted secondary metabolites verified by quantification via UPLC-HRMS

**Fig. 4:**
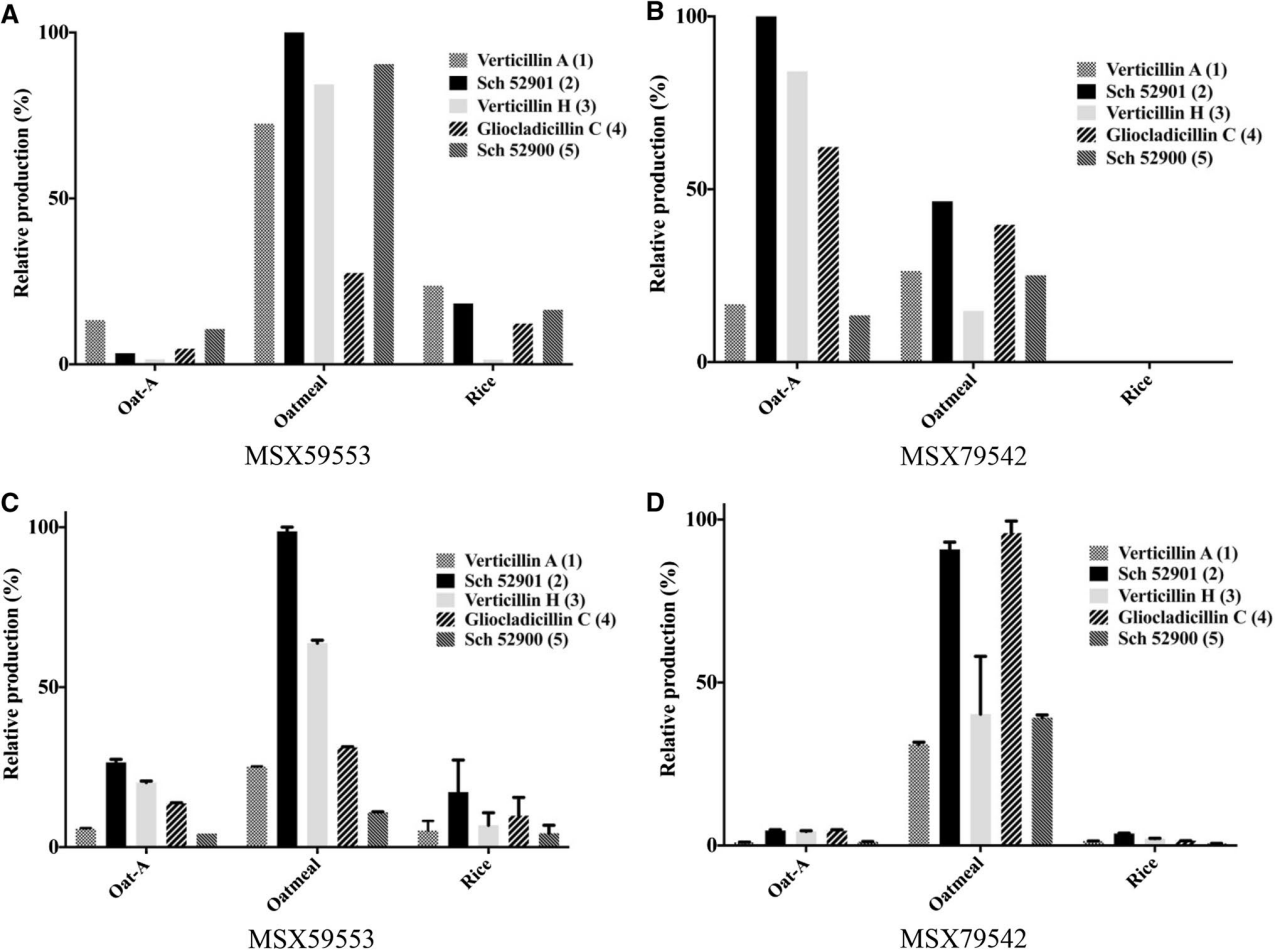
Relative production of verticillin analogues via droplet probe directly from the surface of strains MSX59553 (**a**) and MSX79542 (**b**) grown for 4 weeks. Oatmeal agar showed a higher amount of analogues, likely due to the formation of guttates on the surface of the culture. The same cultures grown on the Petri dishes, and used previously for the droplet probe analysis, were fully extracted and then analyzed via UPLC-HRMS to generate graphs (**c**) and (**d**), which represent the relative total production of verticillin analogues by strains MSX59553 and MSX79542, respectively. The relative percentages were normalized by multiplying the peak areas by the weight of their corresponding organic extracts (Fig S2 Supplementary information). The results from **c** and **d** demonstrate that oatmeal was the best media for total production of verticillin analogues

We next evaluated the production of verticillins on Oat-A vs. readily available substrates, such as breakfast oatmeal and rice (Fig. S3). The UPLC-HRMS analysis of the in situ microextraction on the surface of the three media via the droplet probe were plotted as the relative percentages of the peak normalized according to the compound with highest production (Fig. 4a, b). These results revealed a higher production of the targeted secondary metabolites on oatmeal and Oat-A media compared to the rice. For strain MSX79542, cultures grown on Oat-A showed the highest production, as the fungus expressed guttates (Fig. S6), which have been shown to be concentrated in secondary metabolites [25, 34].

The cultures on Oat-A did not grow as three-dimensional as those grown on rice or breakfast oatmeal. We hypothesized that the three-dimensional character may be beneficial for total verticillin production and thus, the same in situ microextracted Petri dishes were extracted in their entirety, and oatmeal and rice had the highest extract amounts (Fig. S4). The peak areas of the compounds of interest were calculated in their respective total defatted mass of the extract. Then, those numbers were normalized according to the compound of highest production to give a more accurate comparison of the different profiles of verticillin biosynthesis. Regular oatmeal demonstrated the highest amounts of the targeted secondary metabolites (Fig. 4c, d).

To make a more accurate comparison and quantify the amounts of verticillins from the organic extracts of the two strains MSX59553 and MSX79542, cultures were fermented in three biological replicates using rice and oatmeal for comparative purposes. Oat-A was excluded from this comparison because the agar media forces the fungus to grow mainly on the surface of the culture, and that contributed to a low extract weight compared to the other cultures grown for the same amount of time (Fig. S4).

**Fig. 5:**
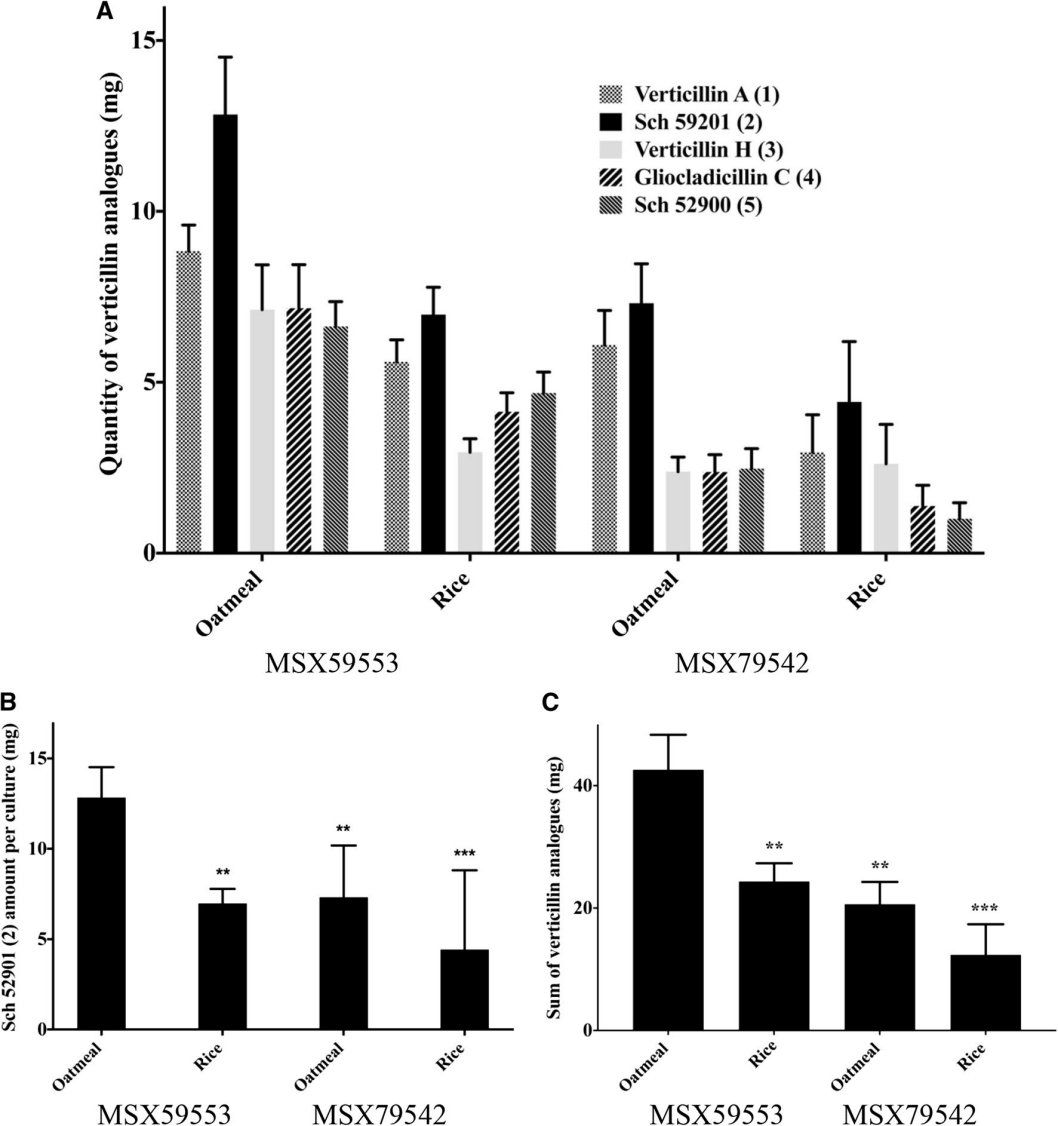
The amounts of the secondary metabolites of interest in mg present in each culture of strains MSX59553 and MSX79542 grown on rice or oatmeal media for 4 weeks in three biological replicates (**a**). The quantity of Sch52901 (**2**), the verticillin analogue produced in the highest amount by the fungal strains, was calculated per flask. The data plotted are mean ± SD of three biological replicates per medium and per strain. The quantity of **2** produced by strain MSX59553 grown on oatmeal showed a significant difference relative to the same strain grown on rice and also compared to strain MSX79542 grown on oatmeal and rice, respectively (**P < 0.004, ***P < 0.0003) (**b**). Similarly, the sum of all verticillin analogues showed a significant difference between the oatmeal culture of strain MSX59553 and the other growth conditions (**P < 0.005, ***P < 0.0003) (**c**)

Verticillin amounts were calculated via UPLC-HRMS calibration curves prepared using purified analogues. To determine the amounts of verticillin analogues produced in mg per flask of growth, the previous numbers were multiplied by the defatted extract mass of each flask. The defatted weights of organic extracts were higher in the strain grown on oatmeal than when it was grown on rice (Fig. S5). Both fungal strains demonstrated the ability to produce higher amounts of verticillin analogues when grown on oatmeal compared to the rice media. Also, using one way ANOVA, strain MSX59553 expressed significantly higher amounts of secondary metabolites when grown on oatmeal than the same strain grown on rice or strain MSX79542 grown on either rice or oatmeal (*P* < 0.005) (Fig. 5b, c).

The extraction and isolation of verticillin analogues from strain MSX59553 grown on a large scale on rice versus that on oatmeal for the same amount of time (5 weeks) was compared. When fermenting on oatmeal, there was a yield of 67.3 mg of verticillins **1–5** per g of extract (with a total weight of pure verticillin analogues isolated being 174.8 mg). Alternatively, when rice was used as a medium, there was 63.3 mg of **1–5** per g of extract (with a total weight of 107.6 mg of purified verticillins). Growth of this fungal culture on oatmeal or rice showed enhanced production of the verticillins, substantially exceeding what had been reported in the literature to date.

### Profile of secondary metabolites based on different growth times

**Fig. 6:**
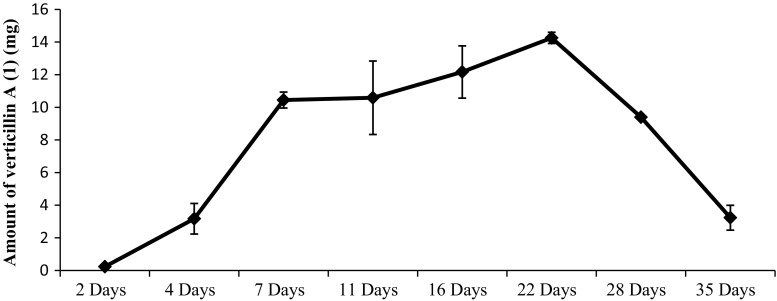
Amount of verticillin A (mg) produced by MSX59553 per flask (with 10 g of oatmeal) during a period of 35 days. The cultures were extracted and then analyzed using UPLC-HRMS. The fungus showed the most production from the 7th to 22nd days of culture before the start of a decline. These data are mean ± SD of two biological replicates, each analyzed in triplicate (*n* = 6)

Duplicate cultures of MSX59553 grown on oatmeal were sampled over 5 weeks to investigate the effect of incubation time on the production of secondary metabolites. The amount of verticillin A was calculated from a calibration curve after injecting 0.025 mg/mL of the extract of each flask. This preliminary amount was multiplied by the total mass of the defatted organic extract to normalize the results (Fig. 6). The production of verticillins increased up to day 7, after which the production of verticillins plateaued by day 22. Interestingly, a decrease of verticillin A was noticed after day 22 until day 35, which was notable since fungal cultures are routinely grown for approximately 4 weeks in laboratory settings. Thus, our study showed that an average of 11 days was enough time for the biosynthesis, which shortened turnaround time for the scaled-up production of the verticillin analogues.

## Conclusions

The verticillins are a class of fungal metabolites whose biological activity against a suite of anticancer assays has stimulated recent interest. However, our challenge was to supply them on a large enough scale, so as to facilitate further preclinical studies. This study showed that fermentation on Quaker oatmeal significantly enhanced the biosynthesis of verticillin analogues in strain MSX59553; whereas we once could isolate about 10 mg after about 5 weeks of growth on rice medium, we can now produce about 20 mg in 11 days on oatmeal medium. To examine a suite of fermentation conditions efficiently, we demonstrated the use of the droplet probe to measure the chemistry of fungal cultures grown in Petri dishes in situ. Parallel studies of extracts from those Petri dishes using a more traditional natural products extraction approach followed by quantitative UPLC-HRMS analysis showed the same trend, serving to further validate the reliability of chemical results from droplet probe analyses. For laboratory-scale production, we are confident that a version of the described procedures could be used to supply verticillins on the single to multi-gram scale. However, further research, potentially exploring a suite of options, ranging from liquid fermentations to semi- and/or total synthesis, may be required should the need for kg quantities arise.

## Electronic supplementary material

Below is the link to the electronic supplementary material.

Supplementary material 1 (DOCX 1555 kb)
