# Supplementary material for: Media studies to enhance the production of verticillins facilitated by in situ chemical analysis
Source: J Ind Microbiol Biotechnol. 2018 Sep 27;45(12):1053–65. doi: 10.1007/s10295-018-2083-8 (PMC6251749; doi:10.1007/s10295-018-2083-8)
Supplement: Supplementary file 1 — Supplementary material 1 (DOCX 1555 kb) [file 10295_2018_2083_MOESM1_ESM.docx]

**Supporting Information**

**Media studies to enhance the production of verticillins facilitated by *in situ* chemical analysis.**

Chiraz Soumia M. Amrine^†^, Huzefa A. Raja^†^, Blaise A. Darveaux^Δ^, Cedric J. Pearce^Δ^ and Nicholas H. Oberlies^†,^*

^†^Department of Chemistry and Biochemistry, University of North Carolina at Greensboro, P.O. Box 26170, Greensboro, North Carolina 27402, United States

^Δ^Mycosynthetix, Inc., 505 Meadowlands Drive, Suite 103, Hillsborough, North Carolina 27278, United States

**Table S1**. Composition of the various agar media evaluated in the current study**.**

**Table S2**. Verticillin analogues observed in fungal strains from the Mycosynthetix (MSX) library.

**Figure S1.** Phylogram of the most likely tree (-lnL = 1766.04) from a RAxML analysis of 67 *Clonostachys* strains based on ITS region (470 bp).

**Figure S2.** *In situ* microextraction via droplet probe directly from the surface of a fungal culture showing the spots where the sampling was performed (black crosses, left). From the central spot towards the edge, the fungal colony goes from the oldest to the youngest. The size of the droplet (~3 to 5 µL) is shown in a black circle (right).

**Figure S3.** Photos of the two strains [MSX59553 (top) and MSX79542 (bottom)] grown on oatmeal agar, oatmeal, and rice (from left to right). Guttates were found only on the oatmeal agar culture of MSX79542 (black arrows). The cultures were grown for 4 weeks at room temperature then analyzed by droplet probe prior to their extraction.

**Figure S4.** The amount of the total defatted extracts of both strains MSX59553 and MSX79542.

**Figure S5.** Total defatted extract weight of strains MSX59553 and MSX79542 grown in flasks in two different media (oatmeal and rice) in three biological replicates.

**Figure S6.** Relative production of verticillin analogues, measured via droplet probe, from the surface of strains MSX59553 grown on YESDA (**A**) and MSX79542 grown on Oat-A (**B**) for 4 weeks. Guttates were observed to contain more analogues then mycelial spots.

**Figure S7.** Relative production of Sch 52901 in cultures of strains MSX59553 on oatmeal grown for 11 days. Each culture received a 790 ppm solution of single amino acids: Trp, Ala, Thr, Glu, Cys; or combinations: (Trp - Cys - Thr), (Trp - Cys -Ala), (Trp - Cys - Glu) and (Trp - Cys - Thr - Ala - Glu). There was also a control culture without amino acid supplementation. The cultures were fully extracted and then analyzed via UPLC-HRMS. The relative percentages were normalized by multiplying the peak areas by the weight of their corresponding organic extracts. The results showed no significant difference between the amino acid enriched media and the control. These data represent means ± SD of two biological replicates, each analyzed in triplicate (n=6).

| Name ( Difco) | \| **Abbreviation** \|  \| \| --- \| --- \| | Composition per 1000 mL Deionized water (DI-H_2_O) |
| --- | --- | --- | --- | --- |
| Malt extract agar | **MEA** | 12.75 g maltose, 2.75 g dextrin, 2.35 g glycerol, 0.78 g gelatin peptone, 15.0 g agar. |
| Potato dextrose agar | **PDA** | 4 g potato starch, 20 g dextrose, 15 g agar. |
| Yeast extract dextrose agar | **YESDA** | 10 g yeast extract, 20 g soy peptone, 20 g dextrose, 15 g agar. |
| Spezieller Nahrstoffarmer agar | **SNA** | 1 g KH_2_PO_4_, 1 g KNO_3_, 0.5 g MgSO_4_.7H_2_O, 0.5 g KCl, 0.2 g glucose, 0.2 g sucrose, 20 g agar. |
| Sabouraud dextrose agar | **SDA** | 5 g peptic digest of animal tissue, 5 g pancreatic digest of casein, 20 g dextrose, 15 g agar. |
| Potato dextrose-mushroom | **PD-mushroom** | 4 g potato starch, 20 g dextrose, 15 g agar, sterilized pieces of *Leucoagaricus* sp. mushroom. |
| Oatmeal agar | **Oat-A** | 60 g oatmeal, 12.5 g agar. |

**Table S1:** Composition of the various agar media evaluated in the current study.

| Strain ID | Verticillin analogues matched via LC-HRMS dereplication |
| --- | --- |
| MSX74391 | Sch52900, Gliocladicillin A, Verticillin A, Verticillin H, Sch52901 |
| MSX71844 | Verticillin A, Sch52900 |
| MSX58124 | Verticillin H, Sch52901, Gliocladicillin C, Sch52900 |
| MSX75296 | Verticillin D |
| MSX75281 | Verticillin D |
| MSX45374 | 11-Deoxyverticillin A, Gliocladicillin, Verticillin H, Sch52901, Gliocladicillin C, Sch52900 |
| MSX70777 | 11-Deoxyverticillin A, Sch52900, Sch52901, Verticillin A, Gliocladicillin A, Gliocladicillin C |
| MSX59553 | Verticillin A, Verticillin H, 11-Deoxyverticillin A, Gliocladicillin C, Sch52900, Sch52901 |
| MSX79542 | Verticillin A, Verticillin H, 11-Deoxyverticillin A, Gliocladicillin C, Sch52900, Sch52901 |

**Table**

**Table S2:** Verticillin analogues observed in fungal strains from the Mycosynthetix (MSX) library. All of these were grown on a rice based medium. Strains MSX59553 and MSX79542 were chosen for further optimization due to their biosynthetic potential to yield a wide variety of desirable analogues.

| 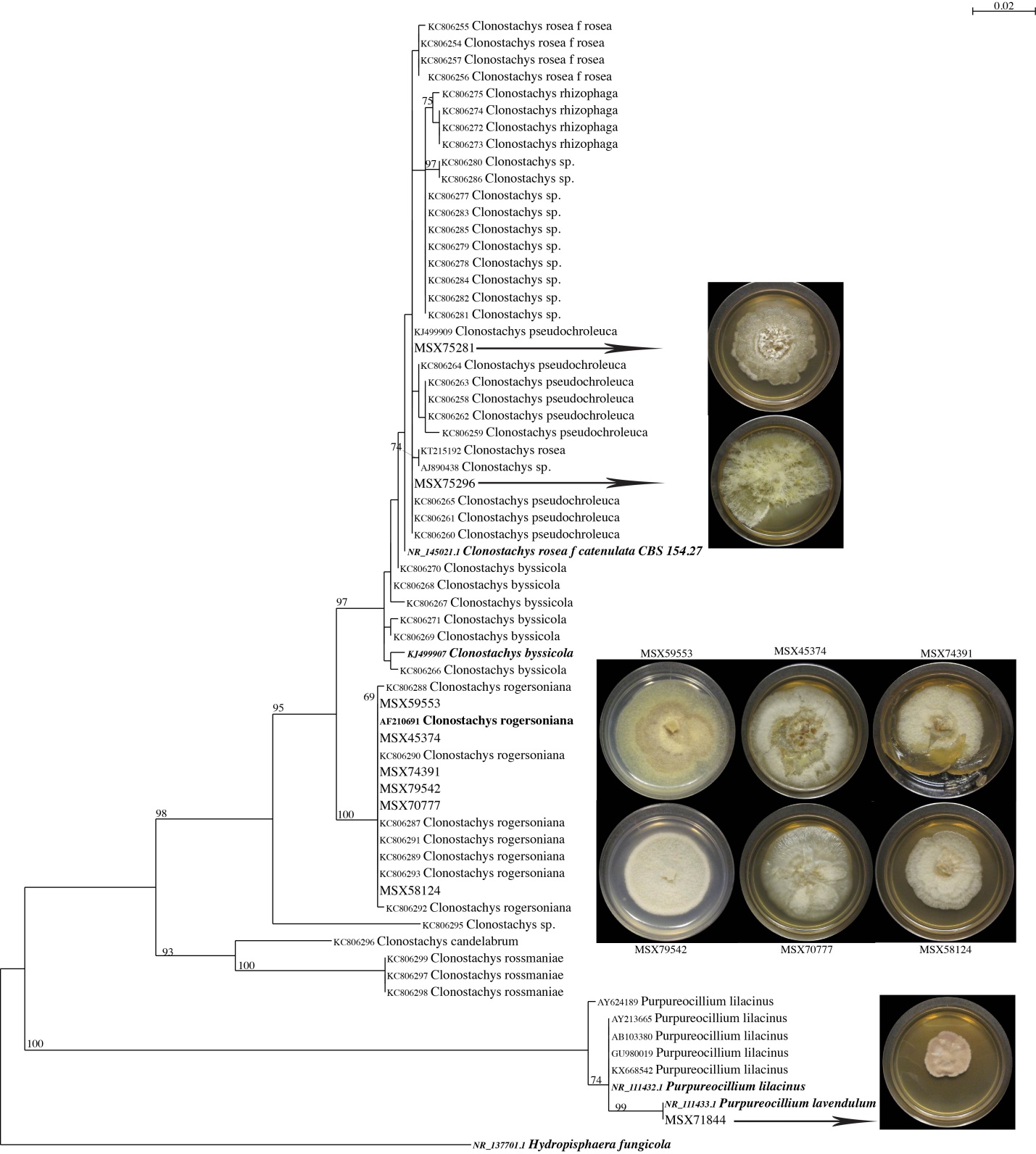 |
| --- |
| **Figure S1:** Phylogram of the most likely tree (-lnL = 1766.04) from a RAxML analysis of 67 *Clonostachys* strains based on ITS region (470 bp). Numbers refer to RAxML bootstrap support values ≥ 70% based on 1000 replicates. Type strains are highlighted in bold italics, authentic strains (bold). Three-week old cultures of MSX strains on Potato Dextrose Agar media are shown on right. Eight strains (MSX75281, MSX75296, MSX59553, MSX45374, MSX74391, MSX79542, MSX70777, MSX58124) are identified as members of the genus *Clonostachys,* while one strain MSX71844 is identified as *Purpureocillium lavendulum.* Bar indicates nucleotide substitutions per site. |

**Table**

| 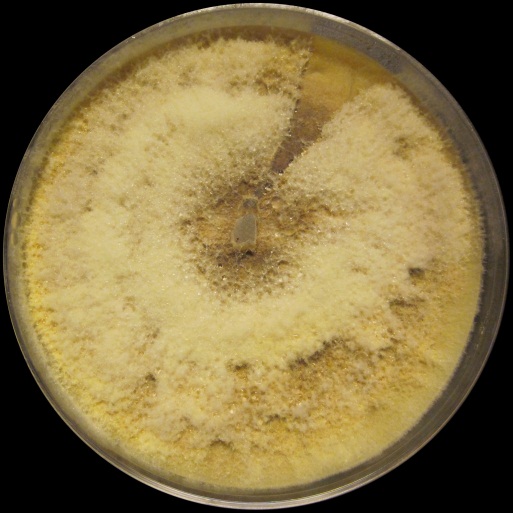 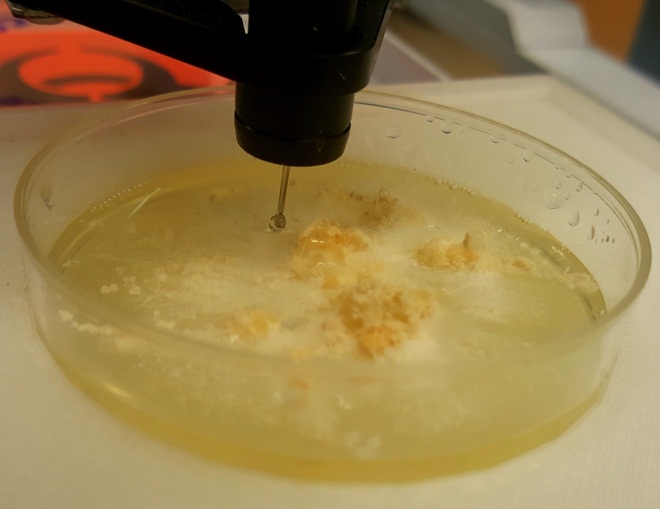 |
| --- |

**Figure S2:** *In situ* microextraction via droplet probe directly from the surface of a fungal culture showing the spots where the sampling was performed (black crosses, left). From the central spot towards the edge, the fungal colony goes from the oldest to the youngest. The size of the droplet (~3 to 5 µL) is shown in a black circle (right).

| **MSX59553** | 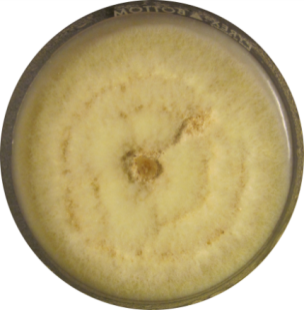 | 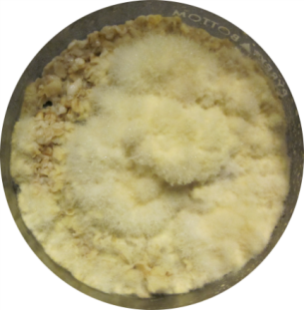 | 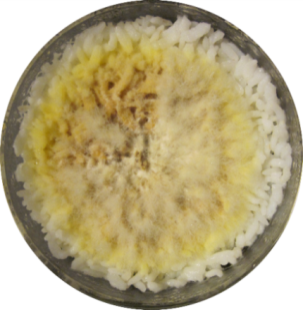 |
| --- | --- | --- | --- |
| **MSX79542** | 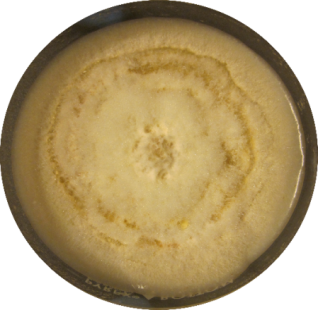 | 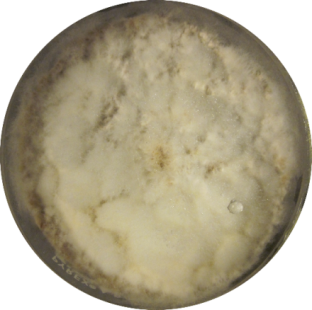 | 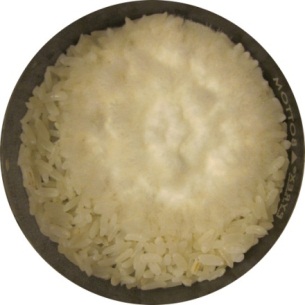 |

**Figure S3:** Photos of the two strains [MSX59553 (top) and MSX79542 (bottom)] grown on oatmeal agar, oatmeal, and rice (from left to right). Guttates were found only on the oatmeal agar culture of MSX79542 (black arrows). The cultures were grown for 4 weeks at room temperature then analyzed by droplet probe prior to their extraction.

**C**

**C**

**Figure S4:** The amount of the total defatted extracts of both strains MSX59553 and MSX79542. The cultures were grown on Petri dishes in three different media (oatmeal agar, oatmeal, and rice).

**Figure S5:** Total defatted extract weight of strains MSX59553 and MSX79542 grown in flasks in two different media (oatmeal and rice) in three biological replicates.

**Figure S6:** Relative production of verticillin analogues, measured via droplet probe, from the surface of strains MSX59553 grown on YESDA (**A**) and MSX79542 grown on Oat-A (**B**) for 4 weeks. Guttates were observed to contain more analogues then mycelial spots.

**Figure S7:** Relative production of Sch 52901 in cultures of strains MSX59553 on oatmeal grown for 11 days. Each culture received a 790 ppm solution of single amino acids: Trp, Ala, Thr, Glu, Cys; or combinations: (Trp - Cys - Thr), (Trp - Cys -Ala), (Trp - Cys - Glu) and (Trp - Cys - Thr - Ala - Glu). There was also a control culture without amino acid supplementation. The cultures were fully extracted and then analyzed via UPLC-HRMS. The relative percentages were normalized by multiplying the peak areas by the weight of their corresponding organic extracts. The results showed no significant difference between the amino acid enriched media and the control. These data represent means ± SD of two biological replicates, each analyzed in triplicate (n=6).
